# Supplementary material for: Structural basis for the activation and ligand recognition of the human oxytocin receptor
Source: Nat Commun. 2022 Jul 18;13:4153. doi: 10.1038/s41467-022-31325-0 (PMC9293896; doi:10.1038/s41467-022-31325-0)
Supplement: Supplementary file 1 — Supplementary Information [file 41467_2022_31325_MOESM1_ESM.pdf]

Supplementary Information

Yann Waltenspühl[1,2], Janosch Ehrenmann[1,3,#], Santiago Vacca[1,#], Cristian Thom[1,#], Ohad Medalia[1] and Andreas Plückthun[1*]

[1] *Department of Biochemistry, University of Zürich, Winterthurerstrasse 190, CH-8057 Zürich, Switzerland.*

[2]*Present address: Novo Nordisk A/S, Novo Nordisk Park 1, DK-2760 Måløv, Denmark*

[3]*Present address: leadXpro AG, PARK innovAARE, CH-5234 Villigen, Switzerland*

#These authors contributed equally: Janosch Ehrenmann, Santiago Vacca, Cristian Thom

[*]*Correspondence and requests for materials should be addressed to A.P. (pluecktun@bioc.uzh.ch*

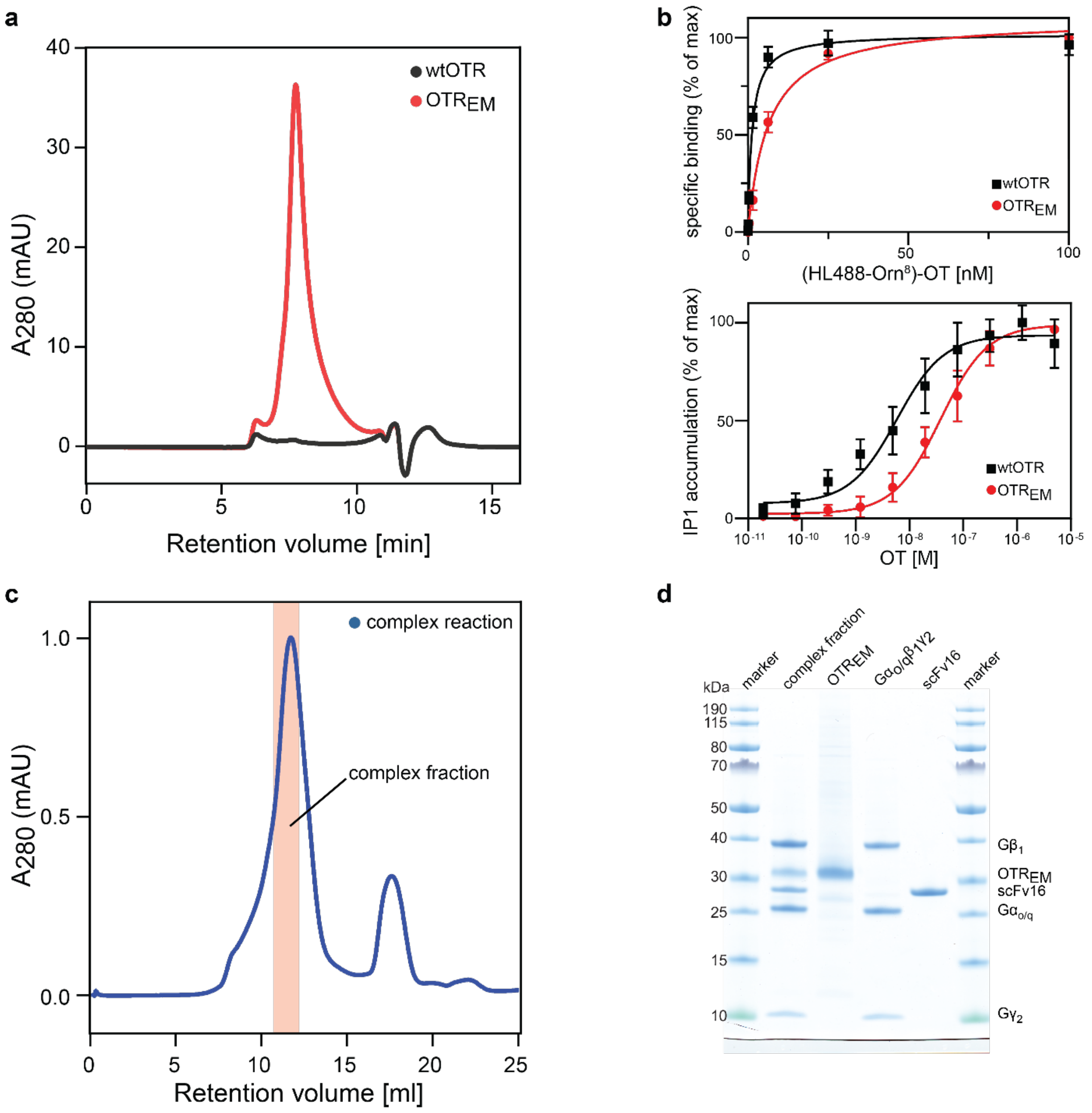

Supplementary Fig. 1 Purification of OTR$_{EM}$ & complex formation.

a Small-scale analytical size-exclusion chromatography (SEC) profiles from initial purifications of wtOTR (black curve) and OTR-D153Y termed OTR$_{EM}$ (red curve). SEC profiles present fair loads. **b** Agonist profiles of wtOTR and OTR$_{EM}$. Dose-response curves were obtained from IP1 accumulation assays, and saturation binding assays were measured by whole-cell ligand binding assays. Saturation binding curves are shown with mean ± standard deviation from six (wtOTR) or three (OTR$_{EM}$) independent experiments performed in triplicates. IP1 dose response curves are shown with mean ± standard deviation from six (wtOTR) or two (OTR$_{EM}$) independent experiments performed in duplicates. Source data are

provided as a Source Data file. **c** SEC profile of the OTR:OT:$G_{o/q}$:scFv16 complex. The red rectangle highlights the pooled fraction used for cryo-EM analysis. **d** LDS-PAGE gel of the pooled complex fraction and the single components.

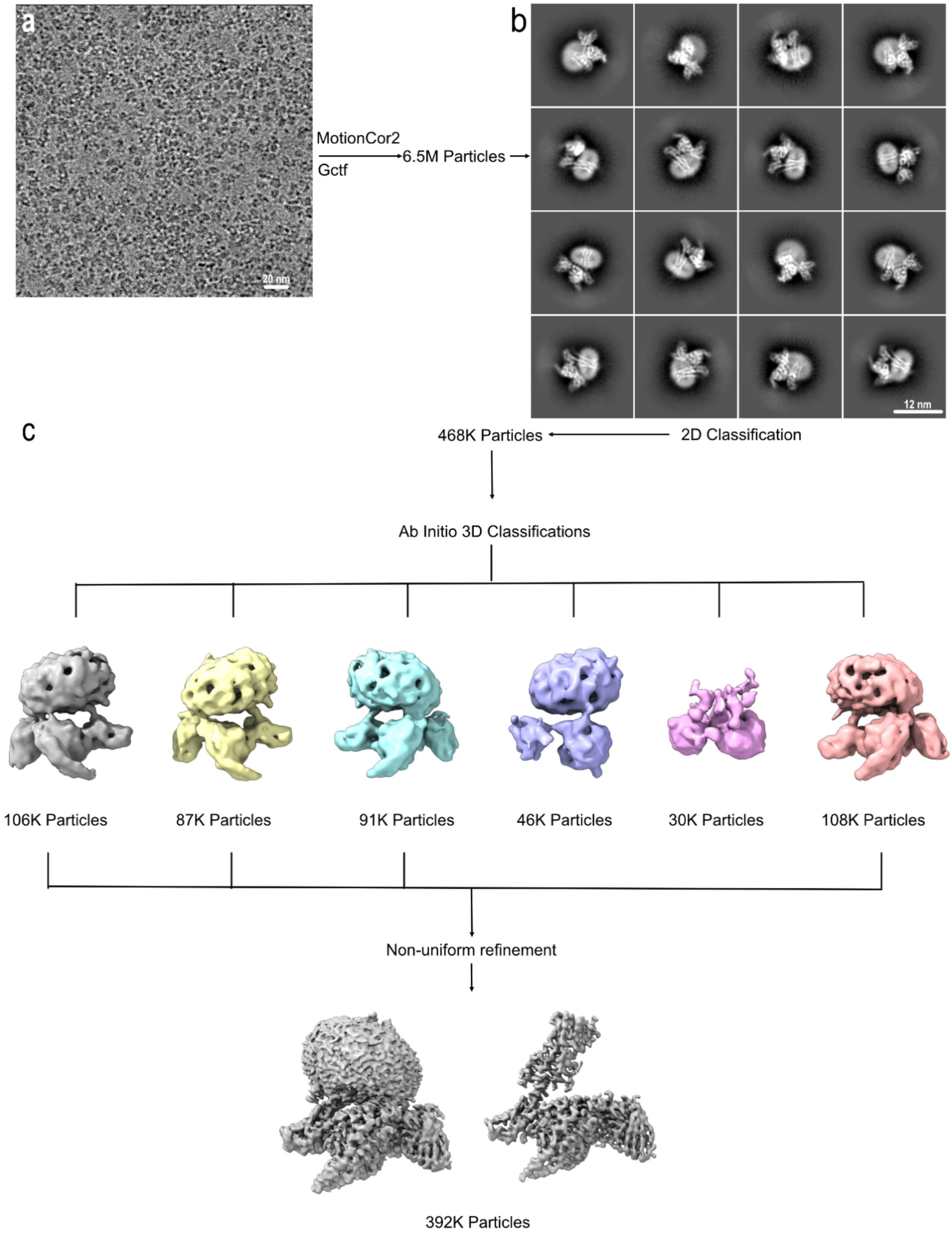

Supplementary Fig. 2 Overview of single-particle cryo-EM data processing.

a A representative cryo-EM micrograph of the 11,667 movie stacks of the OTR:OT:$G_{o/q}$:scFv16 complex. Scale bar, 20 nm. **b** Representative 2D averages showing distinct secondary structure features from different views of the complex. **c** 3D classification workflow and refinement.

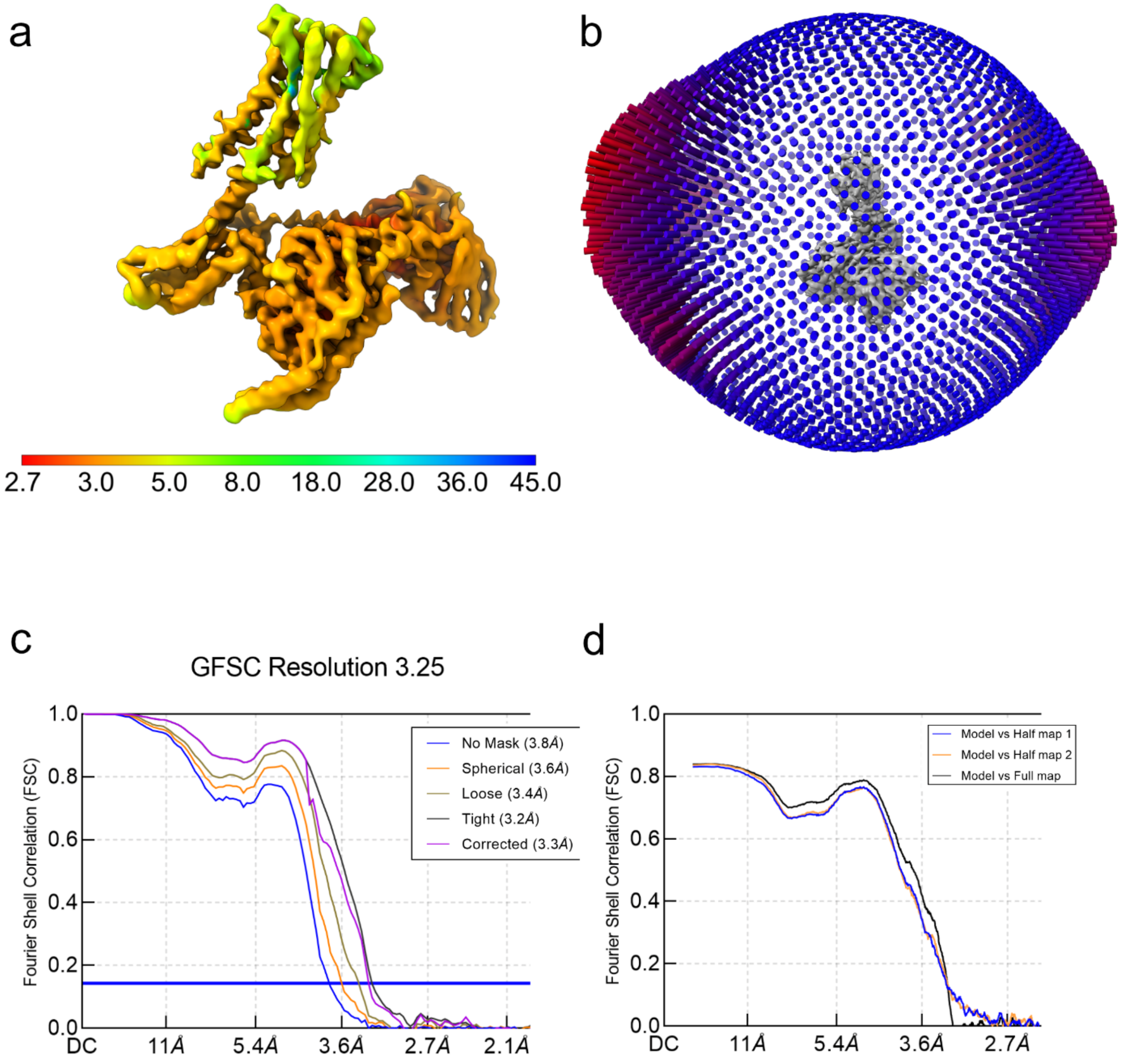

Supplementary Fig. 3 Resolution of the OTR:OT:$G_{o/q}$:scFv16 complex.

a Local resolution analysis of the OTR:OT:$G_{o/q}$:scFv16 complex. **b** Angular distribution of the particle orientations of the OTR:OT:$G_{o/q}$:scFv16 complex. **c** The gold-standard Fourier shell correlation curves for the map of the OTR:OT:$G_{o/q}$:scFv16 complex. **d** For cross-validation, FSC curves of the refined model versus full map (black), refined map versus half map 1 (blue), and refined model versus half map 2 (orange) were calculated.

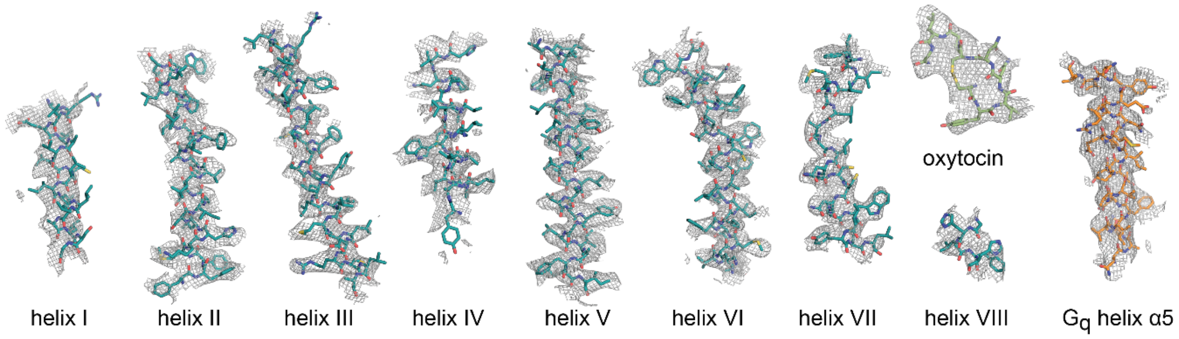

Supplementary Fig. 4 Cryo-EM density within OTR.

Cryo-EM density maps for all OTR transmembrane helices, helix VIII, oxytocin, and the interacting G_q α5 helix of the G protein.

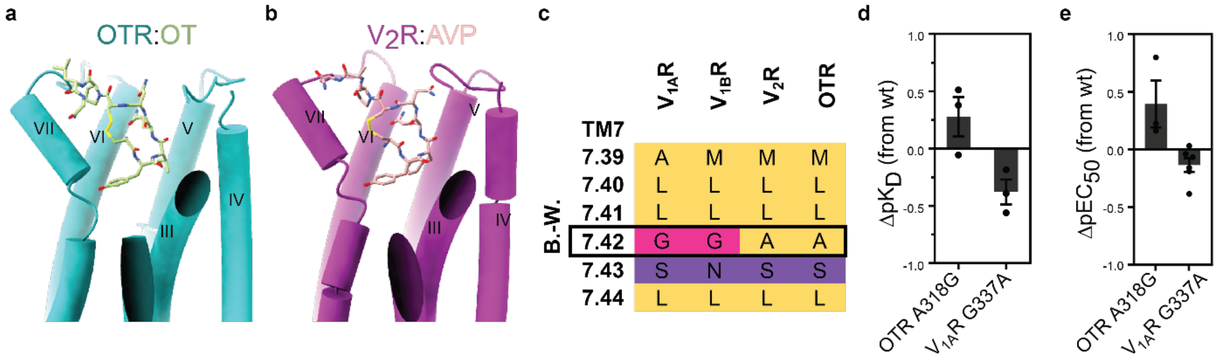

Supplementary Fig. 5 Conserved activation mechanism by oxytocin and vasopressin.

a Cylindrical representation of active OTR:OT complex with close-up on kink in helix VII. **b** Cylindrical representation of active V_2R:AVP complex (PDB ID: 7DW9 [https://www.rcsb.org/structure/7DW9]) with close-up on kink in helix VII. **c** Amino acid sequence alignment of the kink region for all human oxytocin and vasopressin receptors. Amino acid positions are denoted in Ballesteros-Weinstein numbering (B.-W.).**d** OT affinity profiles of OTR and $V_{1A}R$ kink region mutants. Bars represent differences in affinity of the cognate ligand (mean pK_D ± SEM from three independent experiments in triplicates) compared to wtOTR or wt$V_{1A}R$. Source data are provided as a Source Data file. **e** OT IP1 accumulation dose-response curves of OTR and $V_{1A}R$ kink region mutants. Bars represent differences in IP1 accumulation potency of the cognate ligand (mean pEC_{50} ± SEM from three (OTR A318G) or six ($V_{1A}R$ G337A) independent transfections in duplicates) compared to wtOTR or wt$V_{1A}R$. Source data are provided as a Source Data file.

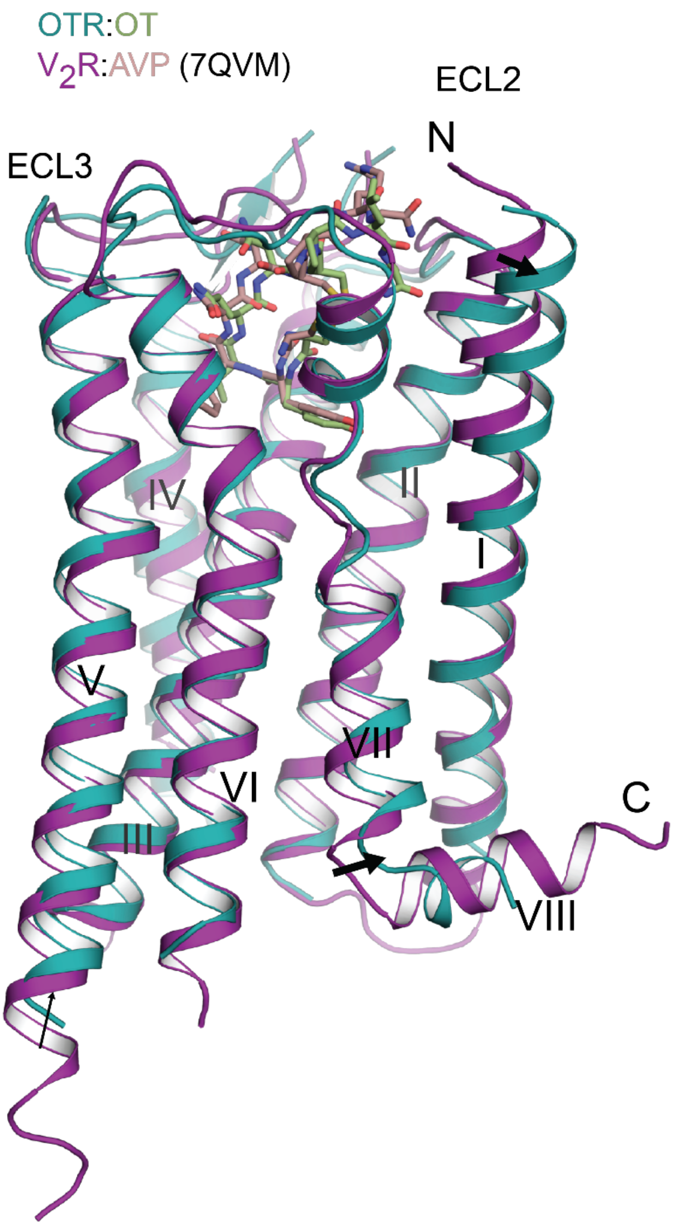

Supplementary Fig. 6 Comparison of the OTR and V$_2$R.

Structural superposition of the OTR:OT with V$_2$R:AVP (PDB ID: 7QVM [https://www.rcsb.org/structure/7DW9], illustrating the main differences between active OTR and V$_2$R. Arrows indicate the main differences in helix positioning and length.

Supplementary Table 1 Single-particle cryo-EM statistics.

	OTR:OT:$G_{o/q}$:scFv16<br>PDB ID: 7QVM
**Data collection**	
Microscope	Titan Krios G3i
Detector	Gatan K3
Energy filter slit width (eV)	20
Magnification	130,000
Voltage (kV)	300
Electron exposure (e^-/Å^2)	63.7
Defocus range (µm)	0.8-2.4
Pixel size (Å)	0.65
Symmetry imposed	C1
Number of Micrographs	11,667
Initial particle images (no.)	6.5 Mio
Final particle images (no.)	392,369
Map resolution (Å)	3.25
FSC threshold	0.143
**Refinement**	
Number of atoms	
All	8,551
Protein	8,482
Ligand	69
Model validation	
CC map vs. model (%)	76
RMSD	
Bond lengths (Å)	0.27
Bond angles (°)	0.640
Ramachandran statistics	
Favored regions (%)	96.4
Allowed regions (%)	3.5
Outliers (%)	0.0
Rotamer outliers (%)	0.0
C-beta deviations (%)	0.0
Clashscore	11.4
MolProbity overall score	1.8

Supplementary Table 2 Effects of mutations on OT-induced IP1-accumulation.

construct	EC_{50} [nM]	ΔpEC_{50}	E_{max} (% of wt)	n
wtOTR	8.4 ± 4	-	100	6
OTR_{EM}	42.9 ± 17	-0.52 ± 0.18	229 ± 29	2
Q92A	222.3 ± 141	-1.72 ± 0.28	12 ± 1	3
Q96A	1540 ± 286.3	-2.75 ± 0.05	68 ± 13	3
K116A	6.1 ± 0.8	-0.36 ± 0.03	48 ± 5	3
Q119A	444.3 ± 192.6	-2.15 ± 0.16	107 ± 33	3
M123A	n.a.	-	(1 ± 1)	3
Q171A	992.5 ± 180	-2.56 ± 0.04	90 ± 12	3
Q171N	39.1 ± 10.2	-1.14 ± 0.1	74 ± 11	3
F175A	3482 ± 857.7	-3.09 ± 0.07	66 ± 12	3
W188A	155.7 ± 62	-1.08 ± 0.18	85 ± 9	2
I201A	89.5 ± 15.4	-1.52 ± 0.12	22 ± 7	3
I204A	n.a.	-	(-2 ± 4)	3
F291A	541.8± 179.4	-1.63± 0.51	28 ± 7	2
F292A	6.1 ± 3.3	0.36 ± 0.62	9 ± 1	2
Q295A	51.3 ± 3.1	-0.63 ± 0.34	32 ± 1	2
L316A	21.9 ± 10.7	-0.21 ± 0.13	18 ± 4	2
A318G	4.6 ± 0.7	0.39 ± 0.2	50 ± 2	3
$wtV_{1A}R$	157.8 ± 27.6	-	100	6
G337A	249.7 ± 90.5	-0.14 ± 0.06	215 ± 15	6

HTRF-based measurements of IP1 accumulation in HEK293T cells expressing wild-type and mutated receptor variants. Activation curves were analyzed by fitting each experiment separately to a three-parameter logistic equation. All values are expressed as mean ± SEM of the indicated number of independent experiments performed in duplicate. n.a., no activation. Source data are provided as a Source Data file.

Supplementary Table 3 Effects of mutations on OT binding.

construct	K_D [nM]	ΔpK_D	B_{max} (% of wt)	n
wtOTR	1.4 ± 0.2	0	100	6
OTR_{EM}	6.4 ± 0.7	-0.6 ± 0.1	252 ± 10	3
A318G	0.9 ± 0.2	0.28 ± 0.17	28 ± 5	3
$wtV_{1A}R$	9.3 ± 2.2	0	100	3
G337A	20.9 ± 0.2	-0.38 ± 0.11	165 ± 23	3

Whole-cell specific saturation binding experiment of fluorescently labelled peptide OT-HL488 to HEK293T cells expressing wild-type and mutated receptor variants. Binding curves were analyzed by fitting each experiment separately to a one-site saturation binding equation. All values are expressed as mean ± SEM of the indicated number of independent experiments performed in triplicate. B_{max} values indicate the amount of functional receptor. Source data are provided as a Source Data file.
