# Supplementary material for: Structural basis for the activation and ligand recognition of the human oxytocin receptor
Source: Nat Commun. 2022 Jul 18;13:4153. doi: 10.1038/s41467-022-31325-0 (PMC9293896; doi:10.1038/s41467-022-31325-0)
Supplement: Supplementary file 3 — Reporting Summary [file 41467_2022_31325_MOESM3_ESM.pdf]

nature portfolio

Corresponding author(s): Andreas Plückthun

Last updated by author(s): May 25, 2022

Reporting Summary

Nature Portfolio wishes to improve the reproducibility of the work that we publish. This form provides structure for consistency and transparency in reporting. For further information on Nature Portfolio policies, see our Editorial Policies and the Editorial Policy Checklist.

Statistics

For all statistical analyses, confirm that the following items are present in the figure legend, table legend, main text, or Methods section.

n/a	Confirmed	
☐	☒	The exact sample size (*n*) for each experimental group/condition, given as a discrete number and unit of measurement
☐	☒	A statement on whether measurements were taken from distinct samples or whether the same sample was measured repeatedly
☒	☐	The statistical test(s) used AND whether they are one- or two-sided<br>*Only common tests should be described solely by name; describe more complex techniques in the Methods section.*
☒	☐	A description of all covariates tested
☒	☐	A description of any assumptions or corrections, such as tests of normality and adjustment for multiple comparisons
☐	☒	A full description of the statistical parameters including central tendency (e.g. means) or other basic estimates (e.g. regression coefficient) AND variation (e.g. standard deviation) or associated estimates of uncertainty (e.g. confidence intervals)
☒	☐	For null hypothesis testing, the test statistic (e.g. *F*, *t*, *r*) with confidence intervals, effect sizes, degrees of freedom and *P* value noted<br>*Give P values as exact values whenever suitable.*
☒	☐	For Bayesian analysis, information on the choice of priors and Markov chain Monte Carlo settings
☒	☐	For hierarchical and complex designs, identification of the appropriate level for tests and full reporting of outcomes
☒	☐	Estimates of effect sizes (e.g. Cohen's *d*, Pearson's *r*), indicating how they were calculated

Our web collection on statistics for biologists contains articles on many of the points above.

Software and code

Policy information about availability of computer code

Data collection	Titan Krios G3i, Gatan K3, EPU software 2.5, Tecan Spark instrument, proprietary software for instrument control
Data analysis	The following software was used in this study: Graphpad Prism 9.2.0, MotionCor2 1.4.0, Gctf 1.06, cryoSPARC 3.0.1, UCSF Chimera 1.15, Pymol 2.5, Coot 0.9.7, MolProbity 4.4, Phenix 1.20.1-4487

For manuscripts utilizing custom algorithms or software that are central to the research but not yet described in published literature, software must be made available to editors and reviewers. We strongly encourage code deposition in a community repository (e.g. GitHub). See the Nature Portfolio guidelines for submitting code & software for further information.

Data

Policy information about availability of data

All manuscripts must include a data availability statement. This statement should provide the following information, where applicable:

- Accession codes, unique identifiers, or web links for publicly available datasets
- A description of any restrictions on data availability
- For clinical datasets or third party data, please ensure that the statement adheres to our policy

Atomic coordinates of the OTR:Go/q:OT:scFv16 complex have been deposited in the PDB under the accession code 7QVM [https://www.rcsb.org/structure/7QVM]. Cryo-EM maps used have been deposited in the EMDB found under code EMD-14180 [https://www.ebi.ac.uk/emdb/EMD-14180]. Source data for ligand binding

and receptor activation experiments are provided with this paper.

Human research participants

Policy information about studies involving human research participants and Sex and Gender in Research.

Reporting on sex and gender	*Use the terms sex (biological attribute) and gender (shaped by social and cultural circumstances) carefully in order to avoid confusing both terms. Indicate if findings apply to only one sex or gender; describe whether sex and gender were considered in study design whether sex and/or gender was determined based on self-reporting or assigned and methods used. Provide in the source data disaggregated sex and gender data where this information has been collected, and consent has been obtained for sharing of individual-level data; provide overall numbers in this Reporting Summary. Please state if this information has not been collected. Report sex- and gender-based analyses where performed, justify reasons for lack of sex- and gender-based analysis.*
Population characteristics	*Describe the covariate-relevant population characteristics of the human research participants (e.g. age, genotypic information, past and current diagnosis and treatment categories). If you filled out the behavioural & social sciences study design questions and have nothing to add here, write "See above."*
Recruitment	*Describe how participants were recruited. Outline any potential self-selection bias or other biases that may be present and how these are likely to impact results.*
Ethics oversight	*Identify the organization(s) that approved the study protocol.*

Note that full information on the approval of the study protocol must also be provided in the manuscript.

Field-specific reporting

Please select the one below that is the best fit for your research. If you are not sure, read the appropriate sections before making your selection.

☒ Life sciences ☐ Behavioural & social sciences ☐ Ecological, evolutionary & environmental sciences

For a reference copy of the document with all sections, see nature.com/documents/nr-reporting-summary-flat.pdf

Life sciences study design

All studies must disclose on these points even when the disclosure is negative.

Sample size	11,667 movies were acquired for structural determination. The cryo-EM maps generated from the movies were sufficient for structural fitting and atomic model building respectively. Ligand binding studies were repeated three times, signalling studies at least two times, both with biologically independent samples (different cell passages or individual transfections) to control for variance normally seen in biological assays, which is comparable to other published studies.
Data exclusions	No data were excluded for ligand-binding assays. Signalling data biased by experimental mistakes (pipetting) were excluded from analysis.
Replication	Ligand binding studies were repeated three times in triplicates, signalling studies at least two times in duplicates, all with biologically independent samples (different cell passages or individual transfections) to control for variance normally seen in biological assays. All attempts of replication were successful. For single particle cryo-EM analysis 6.5 Mio particles were picked from 11,667 micrographs. The eventually resulting structure represents an ensemble average and the experiment was not repeated, as is common and accepted practice in the field.
Randomization	No experimental groups were allocated in cryo-EM and cell-based assays. Thus, randomization was not required for the reported experiments.
Blinding	Blinding was not relevant to the experiments in our study since no subjective allocation was involved.

Reporting for specific materials, systems and methods

We require information from authors about some types of materials, experimental systems and methods used in many studies. Here, indicate whether each material, system or method listed is relevant to your study. If you are not sure if a list item applies to your research, read the appropriate section before selecting a response.

Materials & experimental systems

n/a	Involved in the study
☒	☐ Antibodies
☐	☒ Eukaryotic cell lines
☒	☐ Palaeontology and archaeology
☒	☐ Animals and other organisms
☒	☐ Clinical data
☒	☐ Dual use research of concern

Methods

n/a	Involved in the study
☒	☐ ChIP-seq
☒	☐ Flow cytometry
☒	☐ MRI-based neuroimaging

Eukaryotic cell lines

Policy information about cell lines and Sex and Gender in Research

Cell line source(s)	Sf9, Thermo Fisher Scientific: 11496015; HEK293T/17 cells ATCC: CRL-11268
Authentication	Cell lines are maintained by the supplier. No additional authentication was performed by the authors of this study.
Mycoplasma contamination	The cell lines used in this study were certified and tested negative for mycoplasma contamination by the supplier.
Commonly misidentified lines (See ICLAC register)	No commoly misidentified cell lines were used in this study.
